# HuR-Regulated Extracellular Vesicles Promote Endothelial Cell Remodeling in Pancreatic Cancer

**DOI:** 10.1158/2767-9764.CRC-25-0355

**Published:** 2025-09-03

**Authors:** Jennifer M. Finan, Yifei Guo, Alexandra Q. Bartlett, Kevin Hawthorne, Matthew Reyer, Margaret Haerr, Olayinka Lamikanra, Hen Halamish, Valerie Calvert, Canping Chen, Zheng Xia, Emanuel F. Petricoin, Rosalie C. Sears, Katelyn T. Byrne, Jonathan R. Brody

**Affiliations:** 1 Department of Surgery, School of Medicine, Oregon Health & Science University, Portland, Oregon.; 2 Department of Cell, Developmental and Cancer Biology, Oregon Health & Science University, Portland, Oregon.; 3 Brenden-Colson Center for Pancreatic Care, School of Medicine, Oregon Health & Science University, Portland, Oregon.; 4 Knight Cancer Institute, Oregon Health & Science University, Portland, Oregon.; 5 Molecular and Medical Genetics, Oregon Health & Science University, Portland, Oregon.; 6 Applied Proteomics and Molecular Medicine, George Mason University, Manassas, Virginia.; 7 Biomedical Engineering Department, Oregon Health & Science University, Portland, Oregon.

## Abstract

Pancreatic ductal adenocarcinoma (PDAC) tumors are hypovascular with collapsed and dysfunctional vessels that limit immunosurveillance and contribute to early metastatic events. However, current antiangiogenic therapies have failed in PDAC, highlighting the need to uncover the mechanisms by which cancer cells signal to endothelial cells to increase angiogenesis. Our lab has shown that the tumor-intrinsic RNA-binding protein human antigen R (HuR; *ELAVL1*) plays an important role in reshaping the tumor microenvironment by regulating the stability and translation of cell communication–encoding transcripts. We demonstrate that PDAC-intrinsic HuR influences endothelial cell function in the tumor microenvironment via extracellular vesicle (EV) signaling, an underexplored signaling axis in tumor progression. PDAC EVs contain an HuR-dependent mRNA and protein cargo related to endothelial cell function and angiogenesis. Treating endothelial cells with HuR wild-type (WT) EVs increased the expression of genes involved in barrier function and endothelial cell development and increased their migratory and tube-forming functions. In an immunocompetent mouse model of PDAC, we showed that HuR increased endothelial cell presence and sprouting, while decreasing intercellular adhesion molecule 1 (ICAM-1) expression. Using a genetic EV reporter, we found that ICAM-1 suppression in WT tumors occurred specifically in endothelial cells that had internalized WT EVs, suggesting that this signaling axis modulates endothelial cell behavior *in vivo*. Furthermore, administration of WT EVs rescued impaired HuR-knockout tumor growth, increased endothelial cell abundance, and decreased endothelial cell ICAM-1 expression. Collectively, our data reveal a novel role for HuR as a key mediator of EV signaling to endothelial cells, promoting angiogenesis while restricting endothelial cell leukocyte trafficking behavior.

**Significance:**

Targeting the abnormal pancreatic cancer vasculature remains a significant barrier to immunosurveillance and drug delivery. This study demonstrated that the tumor-intrinsic protein HuR regulates pancreatic cancer EVs, which, in turn, alters endothelial cell behavior. These findings reveal a novel mechanism by which tumor-intrinsic factors shape the vascular microenvironment and suggest that altering EV content could modulate endothelial cell function.

## Introduction

Pancreatic ductal adenocarcinoma (PDAC) remains one of the most challenging solid tumors to diagnose and treat, in part due to its complex and highly desmoplastic tumor microenvironment (TME; refs. 1–3). Embedded within the dense TME, endothelial cells comprise 2% to 10% of the cells within primary PDAC tumors, making them hypovascular when compared with other solid tumors (4, 5). Endothelial cells are involved in angiogenesis, the formation of new blood vessels, leading to increased tumor vasculature. As this new vasculature is often abnormal and characterized by leaky, poorly perfused vessels that promote tumor cell dissemination and metastasis while limiting immunosurveillance, efforts have been made to inhibit angiogenic signaling pathways [e.g., vascular endothelial growth factor (VEGF), platelet-derived growth factor (PDGF), or fibroblast growth factor (FGF); ref. 6]. However, clinical trials targeting these pathways have been unsuccessful (7, 8). These findings highlight the need to strike a balance by inhibiting dysfunctional vasculature while maintaining normal vasculature, to ensure both immunosurveillance and drug delivery (9). Thus, it is important to define how PDAC cells signal to endothelial cells beyond classical growth factor signaling to receptor tyrosine kinases, such as VEGFR, in order to identify therapeutic intervention points to modulate endothelial cell function and improve patient outcomes.

One mechanism by which PDAC cells signal to surrounding cells is via extracellular vesicles (EV), lipid-bound particles that contain nucleic acids, metabolites, and proteins. Seminal work has established the role of PDAC EVs in the metastatic cascade, establishing the premetastatic niche in the liver (10). Notably, liver endothelial cells were among the highest importers of PDAC EVs, suggesting that the PDAC EV–endothelial cell signaling axis plays a role in tumor progression (11). Accordingly, recent studies on the fate of EVs using genetic small EV reporters in mouse models have found that endothelial cells import high levels of PDAC EVs *in vivo* (12, 13). Furthermore, PDAC EV signaling to endothelial cells has been shown to increase endothelial cell proliferation and tube formation *in vitro* (14, 15). Despite these findings, little is understood about the tumor-intrinsic factors crucial for regulating PDAC EV cargoes and their functional impact on recipient cells.

We previously demonstrated that the RNA-binding protein human antigen R (HuR; gene name *ELAVL1*) directly affects the TME composition, in part through the regulation of various cytokines and growth factors (bioRxiv 025.02.07.632847; ref. 16). HuR regulates the production of VEGFA from PDAC cells, indicating a possible intercellular signaling axis with endothelial cells (17). We have shown that HuR posttranscriptionally regulates the translocation, stability, and translation of numerous key stress response proteins, such as PIM1, IDH1, and YAP1, within PDAC cells (bioRxiv 2025.02.07.632847; refs. 18–20). HuR contains three RNA recognition motifs that enable it to bind to a wide array of transcripts containing AU-rich regions, enabling cells to respond to external stressors rapidly (21). In PDAC and other solid tumor types, HuR translocation to the cytoplasm and increased HuR target expression levels are correlated with patient outcomes (bioRxiv 2025.02.07.632847; refs. 16, 22, 23). Furthermore, HuR has been shown to be within colon cancer EVs, supporting metastasis to the lung (24). However, to date, no study has examined the effects of HuR on PDAC EV–mediated cell–cell signaling (25–27). In the current study, we investigated the effect of PDAC-intrinsic HuR on endothelial cells and vascular function via EV signaling. Using RNA sequencing (RNA-seq) and quantitative proteomics, we found that HuR regulates EV cargoes related to endothelial cell biology and that endothelial cell function is altered with wild-type (WT) versus HuR-knockout (KO) EV treatment. Using an orthotopic mouse model of PDAC paired with a genetic EV reporter, PalmGRET, we found that endothelial cell abundance depends on tumor-intrinsic HuR. Furthermore, we found that EVs are imported by endothelial cells *in vivo*, independent of tumor HuR status, and that WT EV import leads to decreased intercellular adhesion molecule 1 (ICAM-1) surface expression in endothelial cells. We showed that WT EVs promote tumor growth, directly increase endothelial cell abundance, and lower ICAM-1 surface expression in HuR-KO tumors. Together, these data define a role of PDAC-intrinsic HuR in regulating tumor EV cargoes that contribute to dysfunctional vascular function *in vivo*.

## Materials and Methods

### Cell lines

Human PDAC cell lines PANC-1 (RRID: CVCL_0480) and MIA PaCa-2 (RRID: CVCL_0428) were obtained from the ATCC and cultured in DMEM supplemented with 10% FBS and 1% penicillin–streptomycin. Cells were authenticated via short tandem repeat analysis at the Oregon Health & Science University (OHSU) Gene Profiling Shared Resource and were routinely tested for *Mycoplasma*. The mouse PDAC cell line (KPC-8069) was a gift from Dr. Michael A. (Tony) Hollingsworth (University of Nebraska Medical Center) and was cultured in DMEM supplemented with 10% FBS and 1% penicillin–streptomycin. CRISPR/Cas9-mediated *ELAVL1* genetic deletion in PANC-1 and KPC-8069 cells was performed by Synthego using scramble or *ELAVL1*-targeted sgRNAs AGA​GCG​AUC​AAC​ACG​CUG​AA (PANC-1, human) and AGA​GCA​AUC​AGC​ACA​CUG​AA (KPC-8069, mouse). As previously described, single-cell clones were isolated and validated for stable KO and proliferation performance (bioRxiv 2025.02.07.632847). Endothelial cells used were human pancreatic microvascular endothelial cells (HPaMEC) purchased from ScienCell Research Laboratories, and human umbilical vein endothelial cells (HUVEC, RRID: CVCL_9Q53) were purchased from ATCC. Endothelial cells were cultured in endothelial cell medium (ScienCell #1001) in flasks coated with fibronectin (2 µg/cm^2^) or Quick Coating Solution (Angio-Proteomie #CAP-01). All the cell lines were cultured in 5% CO_2_ at 37°C in a humidified atmosphere.

### EV isolation

Cells were plated at 2 × 10^6^ KPC-8069 or 3.5 × 10^6^ PANC-1 per 15-cm dish in 20 mL of DMEM supplemented with 10% FBS and 1% penicillin–streptomycin based on the *in vitro* growth rate. The following day, the medium was replaced with DMEM supplemented with 10% EV-depleted FBS. EV-depleted FBS was generated by filtering FBS with an Amicon stirred cell (EMD Millipore UFSC40001) using a 300-kDa ultrafiltration disk (EMD Millipore PBMK07610). After 48 hours, the conditioned media was collected and spun at 2,000 × *g* for 10 minutes to remove cell debris. Next, the media was concentrated to 1 mL using Amicon Ultra-15 Centrifugal Filter Unit (Millipore Sigma #UFC9100) filter columns. Size-exclusion chromatography (SEC) was performed using Izon qEV1 columns with 0.22 µm–filtered PBS, and EVs were collected in pooled fractions 7 to 10. Simultaneously, the cells were trypsinized following conditioned media collection for cell counting and pelleting for EV particle concentration normalization, and controls via immunoblotting.

### VEGF ELISA

VEGF in conditioned media and pooled EV fractions from PANC-1 WT and HuR-KO cells were quantified using the Human VEGF Quantikine ELISA Kit (R&D #DVE00) according to the manufacturer’s instructions. Briefly, 200 µL samples and controls were incubated in microplate strips for 2 hours prior to incubation with the human VEGF conjugate. The substrate solution was added to each well for 20 minutes, the reaction was stopped, and the optical density was determined at 450 nm. A standard curve was used to determine VEGF concentration in each sample.

### Immunoblotting

The cell pellets were washed in PBS and lysed using RIPA lysis buffer (Thermo Fisher Scientific #89900) with a Halt Protease Inhibitor Cocktail (Thermo Fisher Scientific #87786). The protein concentration was quantified for immunoblotting using the Pierce BCA Protein Assay Kit (Thermo Fisher Scientific #23225), and 20 µg samples was prepared in 25 µL with 5× loading buffer. SEC fractions were prepared by suspending 20 µL of SEC sample in 5 µL of 5× loading buffer. Samples were resolved using 10% SDS-PAGE and transferred to a polyvinylidene difluoride membrane (Bio-Rad #1620264). Membranes were incubated with Ponceau, blocked (LI-COR #927-5000), and incubated with primary antibodies (1:1,000) overnight at 4°C in blocking buffer (Supplementary Table S3). The membranes were rinsed, incubated with the secondary antibody (1:20,000), and visualized using an Invitrogen iBright FL1500 Imaging System (RRID: SCR_026331).

### Transmission electron microscopy

The SEC fractions were submitted to the OHSU Multiscale Microscopy Core for sample processing and imaging. The samples were placed on 200-mesh grids coated with carbon and formvar for 3 minutes. The grids were rinsed thrice in water and exposed to 1% (w/v) uranyl acetate for 3 minutes. The grids were blotted dry and imaged on a Thermo Fisher Scientific Tecnai transmission electron microscope operated at 120 kV equipped with an AMT NanoSprint12 camera.

### Fluorescent nanoparticle tracking analysis

The lipid dye Di-8-ANEPPS (Biotium #61012) was prepared by diluting 1:100 in PBS containing 0.05% Pluronic F-127. A measure of 20 µL of each sample was incubated with 1 µL of dye for 15 minutes and immediately diluted with 979 µL H_2_O. Samples were run on the ZetaView Nanoparticle Tracking Analyzer (RRID: SCR_016647) utilizing a 488-nm laser with a 500-nm filter at 11 positions at 25°C. The particle concentration was reported and normalized to the cell count of the donor cells.

### EV RNA extraction

Pooled EV SEC fractions were lysed, and RNA was extracted using the qEV RNA Extraction Kit (Izon #RXT01). According to the manufacturer’s instructions, lysis buffer A was heated at 60°C for 20 minutes prior to use. EV samples were lysed with 900 µL of lysis buffer A and 125 µL of lysis buffer B per 600 µL sample. RNA was isolated using columns and eluted with 50 µL elution buffer. Following isolation, the RNase inhibitor was added to each sample at a final concentration of 1 U/µL (Invitrogen, #AM2682).

### Isobaric-labeling quantitative proteomics

EVs were pelleted by ultracentrifugation at 120,000 × *g* for 2 hours. The samples were ultrasonicated, lysed in 5% SDS and 50 mmol/L triethylammonium bicarbonate, and quantified before reducing, alkylating, and digesting 50 µg of each sample. A measure of 15 µg of peptide from each sample was dried, reconstituted in 25 µL of 100 mmol/L triethylammonium bicarbonate, pH 8, and labeled using six channels (132N–134C) of TMTpro 16plex reagents (Thermo Fisher Scientific). The six labeled samples were pooled and analyzed on an Orbitrap Fusion or Eclipse Tribrid mass spectrometer (Thermo Fisher Scientific) using synchronous precursor selection. The combined labeled peptide digests were separated using 20 online, high-pH reverse-phase fractions, followed by 140-minute low-pH reverse-phase gradient. RAW instrument files were processed using COMET against a canonical human FASTA file, filtered for confident matches using the target/decoy method, and proteins inferred and grouped (28, 29). TMT reporter ions were extracted and combined into protein abundance proxies using an established analysis pipeline. Jupyter Notebooks with an R-kernel were used for quality control and edgeR statistical testing analyses (30). The TMT experiment was normalized using the method of trimmed mean of the M-values, followed by moderated test statistics and modeling. Protein comparisons required Benjamini–Hochberg–corrected *P* values with an FDR <0.1 for a protein to be considered differentially abundant.

### RNA-seq

RNA was isolated from cell pellets using Qiagen RNeasy column purification. RNA isolated from EVs and cells was quantified using a NanoDrop spectrophotometer, and RNA quality was assessed using an Invitrogen Qubit RNA IQ assay. The isolated RNA was shipped to Novogene for poly-A enrichment, library construction, and RNA-seq. Postsequencing analysis involved transcript quantification using Kallisto, which generated the transcripts per million and count matrices. Transcript-to-gene mapping was performed to aggregate transcript counts at the gene level using tximport (31). Differential expression analysis was conducted using the R packages DESeq2 and edgeR to compare groups. For DESeq2, the raw counts were normalized using variance-stabilizing transformation to ensure homoscedasticity across samples. The normalized data were analyzed using a generalized linear model with subject and group as covariates to account for paired experimental designs. Genes with an adjusted *P* value (FDR) <0.05 were considered significantly differentially expressed. The results were annotated using Ensembl IDs and gene names in the biomaRt package. A similar generalized linear model–based pipeline was implemented for edgeR. Count data were normalized using the method of trimmed mean of M-values. Differential expression was assessed via likelihood ratio tests, and significant genes were identified based on FDR thresholds. Additional analyses included generating counts-per-million matrices and exploratory data visualization.

### Reverse-phase protein array

Isolated EVs were shipped to Dr. Emanuel Petricoin for reverse-phase protein array, in which EVs were printed in triplicate on nitrocellulose-coated slides using a Quanterix 2470 Arrayer. Immunostaining was performed by probing each slide with a primary antibody that targeted the target protein. Biotinylated goat anti–rabbit IgG (H + L; 1:7,500, Vector Laboratories) or rabbit anti–mouse IgG (1:10, DakoCytomation) was used as the secondary antibodies. Signal amplification was performed using a tyramide-based avidin/biotin system (DakoCytomation), followed by visualization using streptavidin-conjugated IRDye 680 (LI-COR). The negative controls were stained with a secondary antibody. Total protein was quantified using SYPRO Ruby protein blot staining according to the manufacturer’s instructions (Molecular Probes). The total protein intensity for each sample was calculated by averaging the SYPRO staining intensities of the three replicate spots.

### Single-cell RNA-seq analysis

Single-cell RNA-seq data from 115 PDAC samples were sourced from the Deeply Integrated Single-Cell Omics database (32). A total of 557,304 cells were used to investigate the relationship between *ELAVL1* expression in malignant cells and endothelial cell infiltration. Malignant cells were identified using the R package scATOMIC (v2.0.2) with the parameter “known_cancer_type = PAAD cell” and default settings for other parameters (33). Endothelial cells were defined by the expression of canonical markers, including PECAM1, VWF, and CDH5, based on curated entries from the CellMarker 2.0 database (34). Spearman correlation analysis was used to assess the association between gene expression levels in tumor cells and cell-type infiltration in each sample.

### EV import assays

EVs were labeled with the fluorescent lipid dye PKH67 prepared according to the manufacturer’s instructions and diluted 1:250 in diluent C (Sigma, PKH67GL-1KT). EVs were incubated with the dye suspension for 10 minutes in the dark, suspended over a cell pellet, and immediately centrifuged at 2000 × *g* for 5 minutes to remove excess dye from the solution. PKH67-labeled EVs were suspended in media containing 10% EV-depleted FBS and added to cells plated on eight-well chamber slides (Ibidi #80826). The number of EVs added corresponded to the relative concentration of EVs secreted by cells over 48 hours. This concentration was determined by performing nanoparticle tracking analysis on the isolated EVs to quantify their total number, which was then normalized to the cell count at the endpoint. This consistently yielded approximately 300 EVs per cell. At the endpoint, the media was aspirated, cells rinsed with PBS, and fixed using 4% paraformaldehyde (PFA) for 15 minutes at room temperature. The cells were then rinsed, blocked for 30 minutes, and stained with phalloidin (Biotium #00044, 1:100) and 4′,6′-diamidino-2-phenylindole (DAPI) (1 µg/mL). Cells were imaged at 40× in 4 × 4 field of view tiles on a Zeiss Axio Observer (RRID: SCR_021351). Images were analyzed using ImageJ by creating a mask around the phalloidin signal and measuring the intensity of the EVs.

### Endothelial cell migration assay

A total of 2.5 × 10^5^ HPaMECs or HUVECs were plated on 12-well cell culture inserts (Falcon #353181) that had been coated with fibronectin or a quick coating solution, respectively. PANC-1 WT or HuR-KO EVs were added to the wells below the transwell, and the cells were incubated for 6 hours. The top of the insert was then wiped clean, and the cells that traveled to the lower membrane were fixed with 4% PFA, stained with 0.5% crystal violet, eluted with 5.8 mol/L acetic acid, and the absorbance measured at 590 nm.

### Monolayer permeability assay

A total of 2.5 × 10^5^ HPaMECs and HUVECs were plated in 12-well cell culture inserts (Falcon #353181) coated with fibronectin or quick coating solution, respectively. Cells were incubated with PANC-1 WT EVs or HuR-KO EVs overnight with EV-free endothelial cell media on the bottom of the transwell. The following day, FITC-dextran and EVs were added to the top chamber above the endothelial cell monolayer. After 6 hours, 100 µL of media in triplicate per condition was taken from the bottom of the transwell, plated in a black 96-well plate, and the fluorescence measured (*λ*_excitation_ = 498 nm; *λ*_emmission_ = 517 nm) to assess the amount of FITC-dextran passing through the monolayer.

### Tube formation assay

HPaMECs or HUVECs were treated for 24 hours with PANC-1 WT EVs or HuR-KO EVs. A total of 4 × 10^4^ treated cells were then plated on 15-well polymer coverslip slides (Ibidi 81506) coated with growth factor–reduced Matrigel (Corning #354230). Cells were incubated in a KEYENCE BZ-X fluorescence microscope with live imaging at the Tokai Hit stage and imaged at 4× with phase contrast every hour for 24 hours. The images were analyzed at the 8-hour timepoint at peak tube formation prior to tube degradation using the WimTube image analysis platform.

### Mouse models and treatments

All mouse protocols were outlined in the Institutional Animal Care and Use Committee protocol #00003322 and were approved by the OHSU Department of Comparative Medicine. Subcutaneous tumors were established in 8-week-old female C57BL6 mice from The Jackson Laboratory by injecting 1 × 10^6^ cells suspended in 100 µL of 1:1 cold PBS:Matrigel. The mice were euthanized on day 30, and the tumors were excised and measured. Using the established pancreatic orthotopic survival surgery, 4 × 10^4^ PDAC cells suspended in 20 µL 1:1 cold PBS:Matrigel were injected directly into the tail of the pancreas of 9-week-old male C57BL6 mice from The Jackson Laboratory, as the KPC-8069 cell line was derived from a male mouse. The peritoneum was sutured and the skin closed using wound clips. After surgery, mice were injected with 0.1 mg/kg buprenorphine, provided wet food, and monitored daily for a week for any signs of stress. The mice were weighed weekly and euthanized between 7 and 21 days. For EV treatment, mice were injected intraperitoneally with 100 µL of EVs (1.5 × 10^9^ particles) suspended in PBS on alternating sides every other day for the duration of the study. For functional assessment of mouse vasculature, DyLight 649 I-B_4_ isolectin (Vector Laboratories #DL-1208.5) was administered retro-orbitally 30 minutes prior to euthanasia. Mice were euthanized using CO_2_ followed by cervical dislocation.

### Mouse tumor fixation, embedding, and sectioning

Mouse PDAC tumors were fixed in 4% PFA (Thermo Fisher Scientific #NC9288315) for 24 hours. Tumors for paraffin embedding were then rinsed with 70% ethanol and submitted to the OHSU Histopathology Core for paraffin embedding and sectioning at 5 µm thickness. Tumors for cryosectioning from mice perfused with lectin were transferred from the fixative to 30% sucrose for 24 hours. Tumors were then embedded in optimal cutting temperature, cryosectioned at 8 µm thickness, and stored at −80°C until staining.

### Immunofluorescence staining of mouse tissues

Paraffin-embedded mouse tissue sections were deparaffinized in xylene and rehydrated. Antigen unmasking was performed using a citric acid–based solution, pH 6.0 (Vector Laboratories #H3300250) at a high temperature for 20 minutes. For cryosections, the tissues were rehydrated for 10 minutes in PBS. Tissues were then permeabilized, blocked for 1 hour, and incubated overnight with primary antibody (1:100; Supplementary Table S2). The following day, tissues were rinsed, incubated with secondary antibody (1:500) for 1 hour, quenched for autofluorescence (Vector Laboratories #SP-8400-15), stained with DAPI (1 µg/mL), and mounted (Invitrogen #P36934). Stained tissues were solidified overnight at room temperature and promptly scanned at 20× on a Zeiss Axio Scan.Z1 Slide Scanner (RRID: SCR_020927) at the OHSU Advanced Light Microscopy Core. Images were quantified using QuPath (RRID: SCR_018257). Tumors were annotated, and the percentage of positive staining was calculated using cell detection and object classifiers. Thresholding was determined based on secondary antibody–only control sections.

### Tumor flow cytometry

Tumors were dissociated using gentleMACS, strained through a 40-µm filter, spun at 200 × *g* for 5 minutes, and then incubated with ACK lysis buffer (Gibco #A1049201) for 1 minute. Cells were spun for 5 minutes at 200 × *g* and blocked in Mouse BD Fc Block (BD #553142) diluted 1:100 with fixable LIVE/DEAD (Invitrogen #L23105) diluted 1:500 in PBS for 30 minutes at 4°C. Following blocking, cells were rinsed and resuspended in staining solution with conjugated antibodies diluted in PBS with 2% FBS and 0.5 mmol/L EDTA for 1 hour at 4°C in the dark. Cells were then rinsed twice, strained, resuspended in 200 µL of CountBright beads (Invitrogen #C36950), and run on a Cytek Aurora 5-laser spectral flow cytometer. All experiments included single-color and fluorescence-minus-one controls for gating. For studies leveraging the PalmGRET model, control WT tumors were used to define GFP positivity. Flow cytometry data were analyzed using FlowJo (RRID: SCR_008520). Events were gated on cell size, singlets, and live cells, following the gating strategy outlined in Supplementary Tables S3 and S4. Populations of interest were gated, and cell numbers were calculated as the percentage of live cells, percentage of CD45^+/−^, and cells per gram. Cells per gram were calculated using the equation $cells/g\;=\;\frac{cell\;count\;\times \;total\;beads\;\times \;tissue\;weight\;for\;flow\left(g\right)}{beads\;count}$. The fluorescence intensities were calculated using the geometric mean.

### Ultrasound imaging

Tumor-bearing mice were anesthetized with isoflurane, and their hair was removed. Mice were imaged in 3D color mode using an Ms250s transducer with a 0.1-mm step size on Vevo 2100 (RRID: SCR_015816). Three-dimensional scans were quantified by drawing the tumor margin to determine the percent vascularity on Vevo LAB.

### Transfection and lentiviral transduction

Cell lines were developed to express the PalmGRET EV reporter (pLenti-PalmGRET, Addgene #158221, RRID: Addgene_158221) using a lentivirus produced from transfected LentiX HEK293T cells. After 24 hours of transduction, cells were selected using puromycin dihydrochloride (Sigma #P8833, 5 µg/mL). For HuR re-expression, KPC-8069 cells were transduced with ecotropic retrovirus either carrying the codon-optimized *Elavl1* sequence or empty vector control in the presence of 1 µg/mL polybrene (Millipore Sigma #TR1003G). Cells containing the target constructs were selected using hygromycin B (Millipore Sigma #H3274) and validated for HuR expression by immunoblotting.

### Statistics

All statistical analyses were performed using GraphPad Prism (version 10.1.2, RRID: SCR_002798). *In vitro* data are presented as bar plots with the representative replicate of three shown without single data points as mean ± SD, whereas *in vivo* data are presented as mean ± SE of the mean of one of the two independent experiments. All experiments were performed in biological replicates, defined as independent experiments conducted on distinct biological samples, either as separate cell culture experiments or individual animals. The exact *n* values for the biological replicates are reported in the figure legends. Statistical tests included unpaired two-tailed Student *t* tests to compare two groups and one-way ANOVA for multiple group comparisons with significance defined as *P* < 0.05; **, *P* < 0.01; ***, *P* < 0.001; ns, not significant.

### Data availability

The raw bulk RNA-seq and processed data generated in this study were deposited in the NCBI Gene Expression Omnibus database (GSE303131). The analyzed DESEQ data for RNA-seq are shown in Supplementary Data S1 (WT vs. HuR-KO EV cargo) and Supplementary Data S3 (HPaMECs treated with WT vs. HuR-KO EVs). The raw tandem mass spectrometry data generated in this study have been deposited in the PRoteomics IDEntifications (PRIDE) Archive database, and the accession will be provided upon acceptance of the manuscript for publication with the dataset identifier PXD059674 (35). Proteomic data from WT versus HuR-KO EV cargo are shown in Supplementary Data S2. The remaining data that support the results of this study are available from the corresponding author upon request. The scripts and resources used to generate the analyses have been described in the article.

## Results

### HuR affects PDAC EV cargoes relating to endothelial cell functions

**Figure 1:**
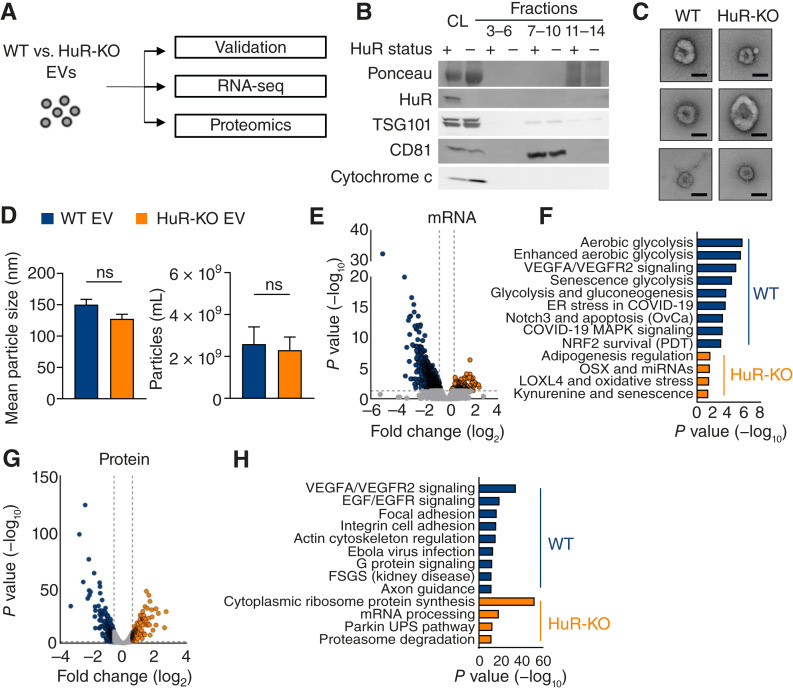
HuR affects PDAC EV cargoes relating to endothelial cell functions. **A,** Schematic of EV isolation from PANC-1 WT vs. HuR-KO cells for EV validation, RNA-seq, and proteomics. **B,** Immunoblot of cell lysates (CL) and SEC fractions 3–6 (pre-EV), 7–10 (EV), and 11–14 (post-EV) from PANC-1 WT (HuR status +) and HuR-KO (HuR status −) cells. Blot probed for total protein (Ponceau), HuR, EV markers (TSG101 and CD81), and cell lysate control (cytochrome c). **C,** Electron microscopy validation of SEC fractions 7–10 (scale bar, 50 nm). **D,** Mean particle size and concentration normalized to final cell number measurements via fluorescent nanoparticle tracking analysis. *P* values were calculated using an unpaired two-tailed Student *t* test (*n* = 4). **E,** RNA-seq volcano plot of differentially abundant genes in WT (blue, left) vs. HuR-KO (orange, right) EVs (*n* = 4). **F,** RNA-seq pathway enrichment analysis highlighting the top nine enriched pathways in WT EVs (blue) and the top four enriched pathways in HuR-KO EV mRNA (orange). **G,** Volcano plot isobaric-labeling quantitative proteomics illustrating the differentially abundant proteins in WT (blue, left) vs. HuR-KO (orange, right) EVs (*n* = 3). **H,** Proteomics pathway enrichment analysis highlighting the top nine enriched pathways in WT EVs (blue) and the top four enriched pathways in HuR-KO EV proteins (orange). ER, endoplasmic reticulum; ns, not significant; PDT, photodynamic therapy; OvCa, ovarian cancer; UPS, unfolded protein response.

PDAC cells rely on cell–cell communication with the prominent stromal compartment within tumors to promote progression. Previous work from our group has highlighted the role of tumor-intrinsic HuR in regulating cell–cell communication through the direct and indirect regulation of cytokines (16). Based on the existing literature highlighting the role of HuR in EV signaling in colorectal cancer, we speculated that tumor-intrinsic HuR would likely play a role in EV signaling (24). To investigate this, we evaluated the effect of HuR-KO on EV secretion and cargo in human PDAC cells (Fig. 1A). We generated PANC-1 mock (scramble sgRNA) and HuR-KO single-cell clones using CRISPR/Cas9 and validated HuR loss at the protein level (Supplementary Fig. S1A and S1B). For subsequent studies, pooled PANC-1 mock, referred to throughout as WT, and PANC-1 HuR-KO clones 1 to 6 were pooled at equal ratios. We isolated EVs from conditioned media collected from PANC-1 WT or HuR-KO cells using SEC, following the MISEV2023 guidelines (36). Next, we validated our EV isolation utilizing protein content, showing a first protein peak in fractions 7 to 10 that contains EVs, followed by a larger peak in fractions 11 to 14, indicative of increased albumin protein levels (Supplementary Fig. S1C). EVs were confirmed to contain the classical markers TSG101 and CD81, while being negative for the cell lysate control cytochrome c (Fig. 1B). Contrary to previous findings in colon cancer, we did not detect HuR in PDAC EVs (Fig. 1B). To evaluate whether SEC completely separated EVs from cytokines, we measured VEGF concentrations in conditioned media before SEC and SEC fractions 3 to 6 (pre-EV), 7 to 10 (EV), and 11 to 14 (post-EV). We found that VEGF was present in the conditioned media and the post-EV fractions 11 to 14 but absent in the EV fractions 7 to 10, illustrating that EVs are separated from cytokines, as previously reported (Supplementary Fig. S1D; ref. 37). Additionally, we observed that HuR-KO significantly decreased VEGF levels in conditioned media, which is in agreement with previous studies (16, 25).

We further validated that membrane-bound EVs were present in SEC fractions 7 to 10 via electron microscopy and fluorescent nanoparticle tracking analysis (Fig. 1C). We found no differences in the size or secretion of the particles isolated using SEC (Fig. 1D). Next, we assessed whether there were different cargoes within the EVs. Upon mRNA-seq of PANC-1 WT vs. HuR-KO EVs, we found that tumor-intrinsic HuR affects mRNA cargoes within PDAC EVs (Fig. 1D). Specifically, PANC-1 WT EVs contained HuR-dependent mRNAs within pathways related to cellular metabolism and endothelial cell biology (Fig. 1E and F; Supplementary Data S1). Given that HuR affects mRNA cargoes within EVs, we performed isobaric-labeling quantitative proteomics analysis on these EVs to identify changes in protein cargoes. Again, we identified a significant number of HuR-dependent proteins (Fig. 1G and H). Pathway analysis revealed that the pathways implicated in endothelial cell function were significantly altered, consistent with the changes in the mRNA cargo (Supplementary Data S2). In agreement with our immunoblot and ELISA results, HuR and VEGF were not within the PANC-1 WT or HuR-KO EVs at the mRNA or protein level. These findings highlight that PDAC EVs contain HuR-dependent cargo distinct from cytokines previously reported to be HuR targets. Additionally, we performed a reverse-phase protein array to better investigate the signaling pathways regulated in a tumor-intrinsic HuR-dependent manner in these EVs. We found that the most differentially abundant proteins and phospho-proteins in EVs were related to endothelial cell biology (Supplementary Fig. S1E and S1F). Together, these data illustrate that tumor-intrinsic HuR, but not within PDAC EVs, regulates the EV cargo, which may regulate endothelial cell function.

**Figure 2:**
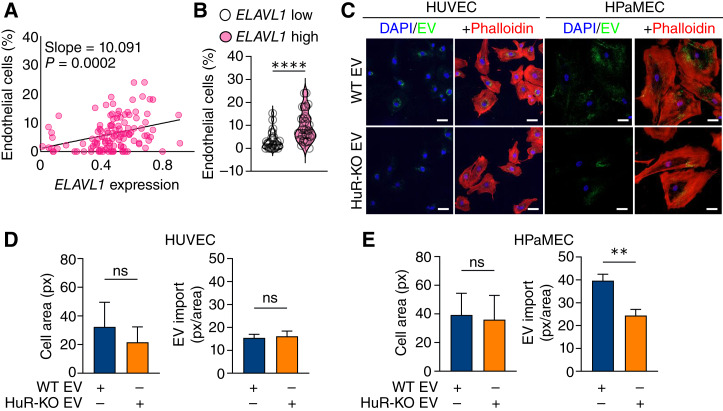
Endothelial cell abundance correlates with *ELAVL1* expression, and endothelial cells import PDAC EVs. **A,** Correlation between *ELAVL1* expression in PDAC cells and the presence of endothelial cells in publicly available patient single-cell RNA-seq. **B,** Within this cohort, a comparison of the endothelial cell percentage in patients with the highest and lowest quartiles of *ELAVL1* expression. **C,** Representative images of HUVECs and HPaMECs treated with PKH67-labeled PANC-1 WT vs. HuR-KO EVs for 4 hours with stained nuclei (blue, DAPI) and cytoskeleton (phalloidin, red; scale bar, 50 µm). **D,** Relative cell area and levels of EV import [pixels (px)/cell area] of HUVECs and (**E**) HPaMECs after 4-hour EV treatment (*n* = 3). *P* values were calculated using an unpaired two-tailed Student *t* test. *, *P* < 0.05; **, *P* < 0.01; ***, *P* < 0.001; ns, not significant.

To determine whether HuR is important for endothelial cell function within the TME in human patients with PDAC, we correlated *ELAVL1* (HuR gene name) expression with the presence of endothelial cells from a publicly available single-cell RNA-seq dataset (32). Strikingly, *ELAVL1* expression in PDAC cells correlated with the abundance of endothelial cells in these data, and there were a significantly higher number of endothelial cells in the top quartile of *ELAVL1*-expressing patients than those in the bottom quartile (Fig. 2A and B). These data suggest that PDAC cell–intrinsic HuR may regulate endothelial cell presence within PDAC tumors, in part via EV signaling.

### PDAC EVs are readily imported by endothelial cells *in vitro*

To assess whether endothelial cells import human PDAC EVs, we optimized *in vitro* EV import studies using the lipophilic dye PKH67 (10, 38, 39). We treated endothelial cells with PKH67 alone or PKH67-labeled EVs for 8 hours and found that cells import a significant level of EVs by 4 hours, after which the lipid signal became diffuse within the cell as the lipids were recycled (Supplementary Fig. S2A). Furthermore, we found that EV import is concentration dependent; thus, for subsequent EV import studies, we treated cells with the concentration of EVs released by PDAC cells in the media over 48 hours, roughly 300 EVs/cell (Supplementary Fig. S2B). Finally, we aimed to ensure that leveraging PKH67 monitored active EV import rather than dye aggregates. To this end, we confirmed that there was no signal in cells incubated with PKH67 alone at 37°C or in those with PKH67-labeled EVs at 4°C after 4 hours (Supplementary Fig. S2C). Using this approach, we observed that HUVECs and HPaMECs imported PDAC EVs (Fig. 2C–E). Interestingly, we found that HPaMECs, but not HUVECs, imported significantly fewer HuR-KO EVs than WT EVs did. These results are consistent with reports that EV import may be a regulated process by which tissue- or cell-specific receptors facilitate EV import through receptor–ligand binding (40–42). Together, these data validate previous findings that endothelial cells import PDAC EVs *in vitro* (12, 14, 15).

### HuR WT EVs directly alter endothelial cell function *in vitro*

**Figure 3:**
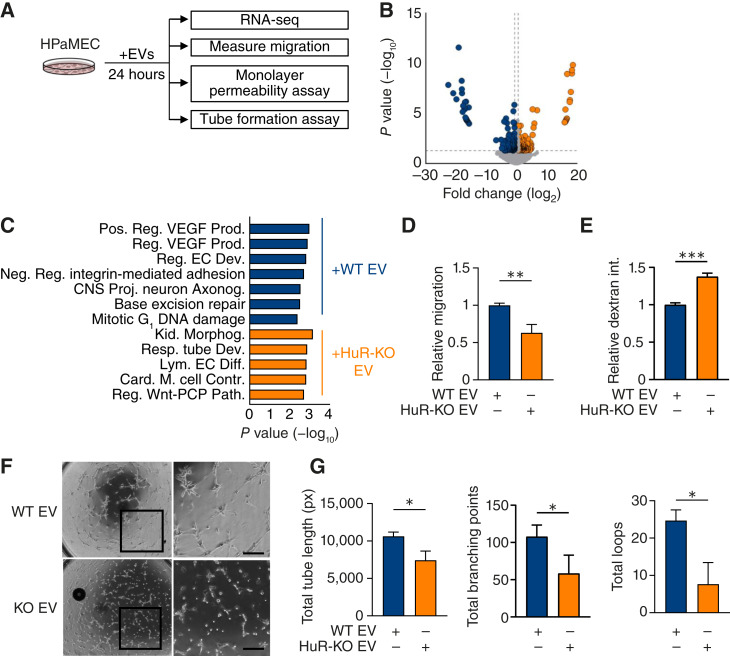
WT EVs directly alter endothelial cell transcriptome, migration, barrier function, and tube formation. **A,** HPaMECs were treated with WT vs. HuR-KO EVs for 24 hours before RNA-seq and phenotypic assessment of migration, monolayer permeability, and tube formation. **B,** Volcano plot of differentially expressed genes in HPaMECs treated with PANC-1 HuR WT (blue, left) vs. HuR-KO (orange, right) EVs for 24 hours (*n* = 3). **C,** Gene Ontology biological process analysis of the top seven pathways enriched in WT EV–treated and the top five pathways enriched in HuR-KO EV–treated HPaMECs (*n* = 3). **D,** Transwell migration of HPaMECs treated with PANC-1 WT vs. HuR-KO EVs over 24 hours and quantified with crystal violet staining (*n* = 3). Functional analysis of HPaMECs treated with media alone, PANC-1 WT, or HuR-KO EVs for 24 hours and monitored for (**E**) monolayer permeability quantified by dextran movement across the endothelial cell monolayer and (**F**) representative phase-contrast tube formation images with full field of view and region of interest (scale bar, 250 µm). **G,** Tube formation quantification for total tube length (px), total branching points, and total loops (*n* = 3). *P* values were calculated using an unpaired two-tailed Student *t* test. *, *P* < 0.05; **, *P* < 0.01; ***, *P* < 0.001; Card. M., cardiac muscle; CNS, central nervous system; Contr., contraction; EC, endothelial cell; Kid., kidney; Lym., lymphatic; Morphog., morphogenesis; ns, not significant; PCP, planar cell polarity; Resp., respiratory; VEGF, vascular endothelial cell growth factor.

Next, we sought to determine whether WT vs. HuR-KO EVs differentially affected endothelial cell function after import using transcriptomic and phenotypic approaches (Fig. 3A). We treated HPaMECs with PANC-1 WT or HuR-KO EVs for 24 hours and performed RNA-seq on the HPaMECs (Fig. 3A–C). We found that transcripts in pathways related to barrier function were significantly altered in WT vs. KO EV–treated endothelial cells (Fig. 3C; Supplementary Data S3). To assess whether these mRNA changes in EV-treated endothelial cells resulted in functional changes, we treated HPaMECs or HUVECs for 24 hours in culture prior to measuring the endothelial cell migration, tube formation, and barrier function (Fig. 3D–G; Supplementary Fig. S3). We observed that HPaMECs and HUVECs migrated more when treated with WT EVs than HuR-KO EVs and that these EV treatments differentially affected barrier function and tube formation. Specifically, we found that WT EV–treated endothelial cells had improved barrier function, which could be either tumor promoting due to decreased immune infiltration or tumor suppressive due to decreased tumor cell intravasation (Fig. 3E; Supplementary Fig. S3B; ref. 43). Furthermore, we found that HuR-KO EV–treated endothelial cells formed fewer tubes than WT EV–treated endothelial cells, as indicated by the decrease in total tube length, number of branching points, and number of loops after 8 hours (Fig. 3F and G; Supplementary Fig. S3C and S3D). These observations demonstrated that HuR-regulated EV signaling has a functional impact on endothelial cells *in vitro*.

### PDAC-intrinsic HuR is necessary for endothelial cell recruitment

Based on our finding that PDAC-intrinsic HuR correlates with the abundance of endothelial cells in patients with PDAC (Fig. 2A), we aimed to determine whether the loss of HuR in mouse models of PDAC would decrease the abundance of endothelial cells *in vivo*. We utilized CRISPR/Cas9 to genetically delete *Elavl1* from PDAC cells derived from the *Kras*^*G12D*^;*Trp53*^*R172H*^;*Pdx1-Cre* (KPC) genetically engineered mouse model as previously reported (bioRxiv 2025.02.07.632847). These mouse PDAC cells contained a mutational profile similar to that of the human PANC-1 PDAC cell line utilized *in vitro* (KRAS^G12D^ and TP53^R273H^). We generated KPC mock cells, referred to as WT cells, and HuR-KO single-cell clones. Three validated HuR-KO clones were pooled for subsequent studies at a ratio of 1:1:1. We implanted immunocompetent C57BL6 mice with subcutaneous WT or HuR-KO KPC cells and allowed the tumors to develop over 30 days. Based on our finding that HuR WT EVs can modulate endothelial cell function *in vitro*, we assessed the presence of endothelial cells in these mouse tumors. We found that HuR tumor cell loss significantly decreased the presence of endothelial cells, as indicated by the endothelial cell marker endomucin (Supplementary Fig. S4A). To ensure that these findings were specifically due to HuR loss, we re-expressed the *Elavl1* sequence via retroviral transduction in HuR-KO cells. This re-expression restored HuR protein levels and led to increased endothelial cell abundance, supporting the specificity of the HuR-dependent phenotype (Supplementary Fig. S4B).

**Figure 4:**
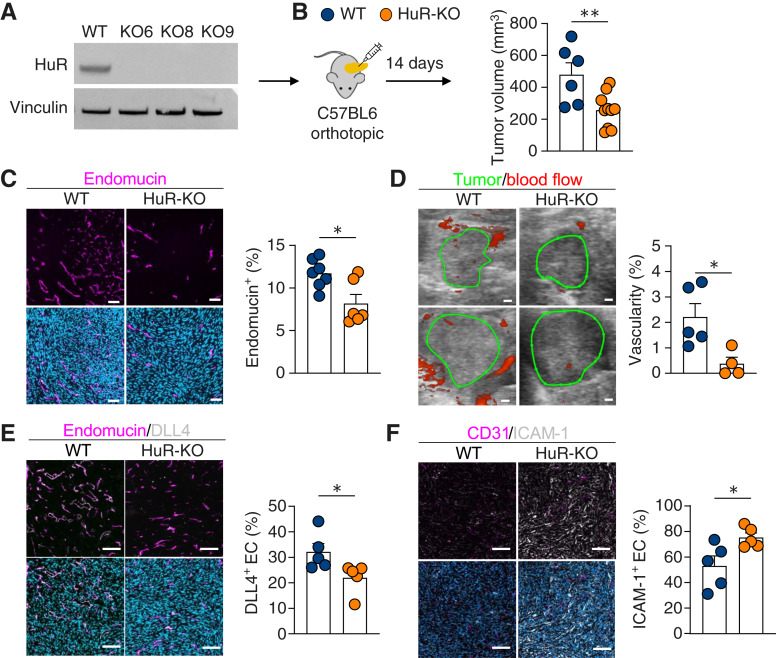
Tumor-intrinsic HuR promotes endothelial cell recruitment and sprouting in PDAC tumors. **A,** Immunoblot of KPC WT and HuR-KO clones 6, 8, and 9 probed for HuR and loading control vinculin. **B,** KPC WT and HuR-KO cells were implanted orthotopically into the pancreas of immunocompetent C57BL6 mice, and tumor volume (mm^3^) was measured after 14 days (WT *n* = 6; HuR-KO *n* = 10). **C,** KPC WT vs. HuR-KO IF staining for endothelial cells (endomucin, magenta) and nuclei (DAPI, teal; scale bar, 100 µm; WT *n* = 7 and HuR-KO *n* = 6). **D,** Ultrasound power Doppler imaging of orthotopic KPC WT and HuR-KO tumors to quantify percent vascularity at 13 days after implantation with tumor border in green and blood flow in red (WT *n* = 5; HuR-KO *n* = 4; scale bar, 1 mm). IF co-staining of KPC WT vs. HuR-KO tumors for (**E**) endothelial cells (endomucin, magenta) and sprouting (DLL4, white) and (**F**) endothelial cells (CD31, magenta) and ICAM-1 (white; scale bar, 100 µm; *n* = 5). *P* values were calculated using an unpaired two-tailed Student *t* test. *, *P* < 0.05; **, *P* < 0.01; ***, *P* < 0.001; ns, not significant.

Next, we validated these findings in an orthotopic pancreatic mouse model by implanting WT and HuR-KO cells into the pancreas of C57BL6 mice and allowing tumors to develop over 14 days (Fig. 4A). As previously reported, HuR-KO orthotopic pancreatic tumors grew smaller than WT tumors in immunocompetent C57BL6 mice (Fig. 4B; bioRxiv 2025.02.07.632847). Similar to the subcutaneous setting, orthotopic HuR-KO tumors had 30% fewer endothelial cells, as quantified using immunofluorescence (IF; Fig. 4C). To test whether the presence of endothelial cells was dependent on tumor size, we implanted more HuR-KO cells based on the *in vitro* growth rate to obtain size-matched WT and HuR-KO tumors at 14 days (Supplementary Fig. S4C). Upon flow cytometry of dissociated tumors for CD31^+^ endothelial cells, we found that there were still significantly fewer endothelial cells in HuR-KO tumors, suggesting that this phenotype depends on tumor-intrinsic HuR rather than tumor size (Supplementary Fig. S4C).

Importantly, this function, in addition to the presence of vasculature, is crucial in affecting tumorigenesis; thus, we aimed to assess whether the vasculature in WT versus HuR-KO tumors functioned similarly (9). We performed ultrasound imaging on WT versus HuR-KO tumor-bearing mice 13 days after implantation using the power Doppler 3D mode to measure percent vascularity, indicative of the relative vascular density within tumors. In accordance with the decreased endothelial abundance via IF staining, we found that there was a decrease in percent vascularity in HuR-KO tumors compared with that in WT tumors (Fig. 4D). Next, we retro-orbitally injected fluorescently labeled lectin to label the functionally perfused vasculature within the tumors and assessed the percentage of lectin^+^ vasculature by staining for endomucin. We observed that both WT and HuR-KO tumors had approximately 50% functionally perfused vasculature (Supplementary Fig. S4D). Together, these data indicate that although HuR-KO tumors have less overall vasculature, the proportion of perfused vasculature is equal to that in WT tumors.

To further interrogate the function of the vasculature within these tumors, we stained for a marker of endothelial cell sprouting, DLL4. We found that there were 45% more sprouting endothelial cells in WT tumors than in HuR-KO tumors, indicating increased vascular remodeling (Fig. 4E). Next, we evaluated the surface expression of ICAM-1, an important cell surface glycoprotein that serves as a scaffold for leukocyte binding, aiding transendothelial cell migration (44, 45). We observed that ICAM-1 surface expression in endothelial cells increased in HuR-KO tumors, suggesting an increase in inflammatory activation in HuR-KO tumor endothelial cells (Fig. 4F). Taken together, these data suggest that PDAC-intrinsic HuR increases endothelial cell abundance and sprouting while decreasing the ability of the vasculature to aid leukocyte trafficking within the TME.

### PDAC HuR affects endothelial cells *in vivo* directly via EV import

**Figure 5:**
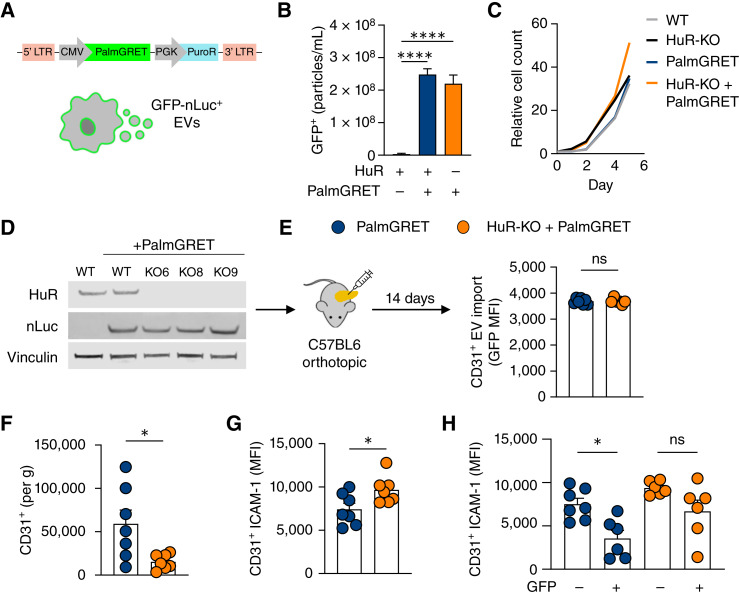
Tumor-intrinsic HuR directly alters endothelial cell function via EV signaling. **A,** Schematic of the PalmGRET reporter labeling the inner leaflet of all membranes with GFP-nLuc via integration of a palmitoylation sequence. The construct for the PalmGRET EV reporter labels the inner leaflet of all cellular membranes and secreted membranous particles. **B,** PalmGRET-expressing cells produce GFP^+^ EVs detected by fluorescent nanoparticle tracking analysis (*n* = 3). **C,** Cell growth of PalmGRET-expressing KPC cells over 6 days. **D,** Immunoblot of cell lysates utilized for mouse studies probed for HuR, nLuc, and loading control vinculin. **E,** PalmGRET-expressing cells were orthotopically implanted into C57BL6 mice, and tumors were harvested at 14 days for flow cytometry. Levels of EV import (GFP MFI) in endothelial cells (CD45^−^CD31^+^) were equal in WT and HuR-KO tumors (*n* = 7). **F,** Total endothelial cell (CD45^−^CD31^+^) presence as quantified by cells per gram of tumor in WT vs. HuR-KO tumors (*n* = 7). **G,** ICAM-1 geometric mean surface expression of ICAM-1 in endothelial cells (CD45^−^CD31^+^) of WT vs. HuR-KO tumors (*n* = 7). **H,** ICAM-1 geometric mean surface expression (MFI) of ICAM-1 in endothelial cells of WT vs. HuR-KO tumors that have (GFP^+^) or have not (GFP^−^) imported PDAC EVs (*n* = 7). *P* values were calculated using an unpaired two-tailed Student *t* test or an ordinary one-way ANOVA (**H** only). *, *P* < 0.05; **, *P* < 0.01; ***, *P* < 0.001; ns, not significant. MFI, mean fluorescence intensity.

EV signaling dynamics are affected by many physiologic factors, including matrix stiffness and pH, which differ considerably between *in vitro* cultures and PDAC tumors (46–49). To study the cross-talk between HuR-regulated EVs and the TME, we employed an established genetic EV reporter, PalmGRET, which incorporates GFP-nLuc into the inner leaflet of cellular lipid bilayers via a palmitoylation sequence (Fig. 5A; refs. 50, 51). By leveraging this model, we determined whether lipid-bound particles, including EVs derived from WT and HuR-KO PDAC cells, were trafficked to endothelial cells within the TME. Importantly, this model relies on the endogenous production of EVs from tumor cells within a physiologic setting with tumor-related stressors rather than the administration of exogenously produced EVs. We validated that this reporter leads to the production of GFP^+^ and nLuc^+^ EVs utilizing two independent techniques (Fig. 5B; Supplementary Fig. S5A). Furthermore, the addition of this construct did not alter the *in vitro* growth of these cells; notably, *in vivo* tumor size was altered, yet not significantly (Fig. 5C; Supplementary Fig. S5B). This may be due to the immunogenicity of exogenous proteins such as GFP and nLuc, a caveat to utilizing any currently available genetic EV reporter (52). We generated WT and HuR-KO cells expressing the PalmGRET reporter and sorted them using FACS to ensure that they had an equivalent GFP signal (Supplementary Fig. S5C), as confirmed in each subsequent experiment via immunoblotting for nLuc (Fig. 5D). Next, these cells were used to assess which stromal cells imported PDAC WT vs. HuR-KO EVs *in vivo* by establishing orthotopic pancreatic tumors in immunocompetent mice (Fig. 5E).

We sought to determine the levels of PDAC EV import by endothelial cells within the TME. To address this, we performed flow cytometry on tumors, stained for stromal cell markers, and evaluated the GFP intensity (Supplementary Tables S3 and S4). As a control and to determine gate placement for GFP positivity, each study contained mice bearing KPC WT cells without the PalmGRET reporter. Stained mixes of all WT and PalmGRET tumors in each experiment were used to define GFP positivity (Supplementary Fig. S5D). GFP geometric mean fluorescence intensity within endothelial cells (CD45^–^CD31^+^) in WT and HuR-KO tumors was similar, indicating that endothelial cells within both tumors import PDAC EVs, irrespective of PDAC cell–intrinsic HuR (Fig. 5E). These findings are consistent with our *in vitro* data, demonstrating EV import by both HUVECs and HPaMECs (Fig. 2C). Although there were differences in WT and HuR-KO EV import by HPaMECs (a more relevant model because of their pancreatic origin), there was no difference in our mouse model. This highlights the importance of studying EV signaling *in vivo*, in which complex factors beyond simple receptor–ligand interactions likely contribute to these observed effects. Despite the variability in EV internalization *in vitro*, both cell types exhibited similar responses to the HuR-dependent EV cargo, suggesting convergence in downstream signaling pathways that can be monitored in the mouse model. In concordance with previous studies, we observed that other cells, including cancer-associated fibroblasts (CAF) and immune cells within the TME, imported PDAC EVs in both WT and HuR-KO tumors (Supplementary Fig. S5E). Importantly, the high GFP signal in macrophages may be indicative of both EV import and phagocytosis, as these two events cannot be distinguished based on GFP signal alone. Our data are in agreement with previously published studies in which CAFs were the major importers of PDAC EVs (13). Together, these data suggest that the tumor-promoting role of HuR via EV signaling occurs because of the impact of EV cargo on endothelial cells, rather than the rates of EV import.

In addition to EV import rates, we calculated the number of endothelial cells per gram of tumor and found that in accordance with previous studies, HuR-KO tumors had significantly fewer endothelial cells (CD45^−^CD31^+^; Fig. 5F). Furthermore, we observed that ICAM-1 surface expression on endothelial cells was lower in WT tumors than in HuR-KO tumors, which is in agreement with our findings in the KPC model without PalmGRET (Fig. 5G). To test the causal relationship between EV import and endothelial cell ICAM-1 surface expression, we compared ICAM-1 surface expression in endothelial cells that had imported a PDAC EV (GFP^+^) versus those that had not (GFP^−^). We found that within WT tumors, endothelial cells that had imported a PDAC EV had significantly lower ICAM-1 surface expression than endothelial cells that had not imported a PDAC EV (Fig. 5H). However, endothelial cells in HuR-KO tumors showed elevated ICAM-1 surface expression, irrespective of whether they were GFP^+^. These data suggest that HuR-dependent cargoes within WT EVs lead to decreased ICAM-1 surface expression in endothelial cells and decreased endothelial cell leukocyte trafficking.

### Tumor-intrinsic HuR promotes tumor growth and endothelial cell abundance via EV signaling

**Figure 6:**
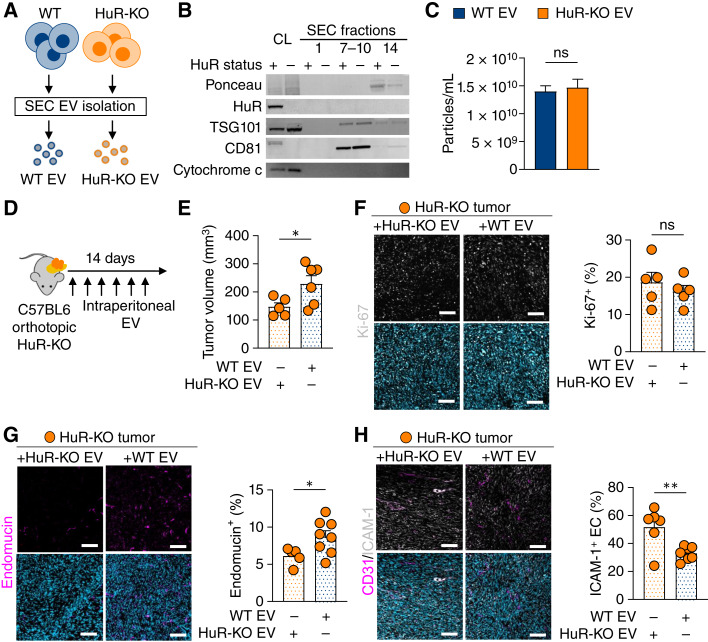
HuR WT EVs are tumor promoting and directly decrease ICAM-1 expression on endothelial cells. **A,** Schematic of EV isolation from KPC WT and HuR-KO cells via SEC. **B,** Immunoblot of cell lysates (CL) and SEC fractions 1, 7–10 (EV containing), and 14 from KPC WT (HuR status +) and HuR-KO (HuR status −) cells. Blot probed for total protein (Ponceau), HuR, EV markers (TSG101 and CD81), and cell lysate control (cytochrome c). **C,** Particle size concentration normalized to final cell number measurements via fluorescent nanoparticle tracking analysis. (*n* = 4). **D,** Schematic of pancreatic orthotopic implantation of KPC KO cells into C57BL6 mice following intraperitoneal injection of WT vs. HuR-KO EVs every other day for 14 days and (**E**) tumor volume (mm^3^) after 14 days (WT EV *n* = 6; HuR-KO EV *n* = 5). IF staining for (**F**) proliferation (Ki-67, magenta; *n* = 5), (**G**) endothelial cells (endomucin, magenta; WT EV *n* = 5 and HuR-KO EV *n* = 8), and (**H**) co-staining of endothelial cells (endomucin, magenta) and ICAM-1 (white; WT EV *n* = 6 and HuR-KO EV *n* = 7; scale bar, 100 µm). *P* values were calculated using an unpaired two-tailed Student *t* test. *, *P* < 0.05; **, *P* < 0.01; ***, *P* < 0.001; ns, not significant.

To directly test the role of WT EVs in mediating endothelial cell function *in vivo*, we investigated whether administering WT or HuR-KO EVs into HuR-KO tumor-bearing mice would alter the tumor vasculature. First, we isolated KPC WT and HuR-KO EVs using SEC (Fig. 6A; ref. 36). EVs were confirmed to contain the classical markers TSG101 and CD81, while being negative for HuR and cytochrome c, regardless of HuR status (Fig. 6B). Similar to our findings using human PANC-1 PDAC cells, there was no difference in the size or secretion of EVs between WT and HuR-KO KPC cells (Fig. 6C). Previous studies administering exogenously produced EVs relied on both intraperitoneal and intravenous injections of EVs (10, 11). To determine the impact of WT versus HuR-KO EVs in HuR-KO tumor-bearing mice, we tested whether PDAC EVs would be imported by PDAC and stromal cells in the TME when administered intraperitoneally or intravenously (Supplementary Fig. S6A). We assessed EV import across tissues in mice and found that both intraperitoneal and intravenous injections led to significant EV import within PDAC tumors. Thus, we proceeded with intraperitoneal injection to avoid confounding factors with intravenous injection, such as altered tumor vasculature in our mouse model.

We implanted KPC HuR-KO cells into the pancreas of immunocompetent mice and administered WT or HuR-KO EVs intraperitoneally every alternate day for 14 days (Fig. 6D). We found that HuR-KO tumors treated with WT EVs were significantly larger at the endpoint, suggesting that tumor-intrinsic HuR plays a tumor-promoting role via EV signaling (Fig. 6E). Importantly, HuR was not present within WT EVs, indicating that HuR has a tumor-promoting effect, likely through cargoes that are different in EVs derived from WT vs. HuR-KO cells (Figs. 1B and 6E). Furthermore, we found that WT EV and HuR-KO EV–treated tumors had equivalent Ki-67 positivity using IF staining, suggesting that the differences in tumor size were not due to changes in proliferation but due to alterations in the TME (Fig. 6F). Moreover, WT EV–treated HuR-KO tumors showed increased endothelial cell abundance, highlighting the direct role of PDAC EVs in increasing endothelial cells within the TME (Fig. 6G). Of the endothelial cells present in WT EV–treated HuR-KO tumors, ICAM-1 surface expression was decreased, supporting our hypothesis that PDAC EVs directly decreased endothelial cell ICAM-1 surface expression (Fig. 6H). Overall, these findings highlight an important role for HuR in regulating the function of the vasculature within the TME, which can impact tumor progression, immunosurveillance, and response to therapy (53, 54).

## Discussion

Intercellular communication has long been understood to play a role in many facets of tumor progression, enabling genetically mutated cancer cells to co-opt normal tissues (1, 2, 55). More recently, EV signaling has been recognized as a coordinated signaling mechanism within and across tissues, with improvements in genetic EV reporters to study this signaling axis *in vivo* (13, 50, 56, 57). In PDAC, EVs have been shown to be crucial in establishing a premetastatic niche and have the potential to play a role early in the metastatic cascade via signaling to endothelial cells and CAFs within the TME (10, 11, 13). However, little has been done to understand the key cargoes and regulators of these cargoes in PDAC EV signaling. Our group and others have established the role of the aberrantly regulated RNA-binding protein HuR in numerous cell-intrinsic stress-adaptive processes and more recently in cell–cell signaling within the TME (bioRxiv 2025.02.07.632847; ref. 16). Herein, we aimed to determine the role of PDAC-intrinsic HuR in EV signaling in the TME and found that PDAC EVs are tumor promoting in an HuR-dependent manner, likely owing to their impact on endothelial cell function.

We found that HuR-KO cells produce EVs at the same rate and size; however, there are significant changes in mRNA and protein cargos, both of which can induce functional changes in recipient cells. These findings were EV specific, as we validated that our isolation excluded cytokines such as VEGF. Notably, we found that HuR itself is not present within PDAC EVs; rather, HuR regulates cargoes within EVs. We found that both mRNAs (e.g., *DKK1*, *RHOA*, *SHC1*, *AKT1*, and *JUNB*) and proteins (e.g., ITGB1, RAC1, DKK1, and ENG) related to metabolism and endothelial cell function were the most affected. We further found that EV biogenesis proteins remained unchanged with HuR status, supporting our finding that HuR did not change EV secretion rates. We and others have shown that PDAC EVs are imported by endothelial cells *in vitro* and *in vivo* (11–15). Our findings corroborate the work performed in two other independent genetic EV tracking models, in which EVs are imported by specific cell types rather than equally across all cells in the TME (13). However, by leveraging the PalmGRET model, we were able to track a broad, heterogeneous population of EVs rather than a subset of lipid-bound particles (56). Although there are changes in the mRNA and protein cargoes with the loss of tumor-intrinsic HuR, there are no changes in *in vivo* PDAC EV import. This suggests that the molecules involved in PDAC EV import are HuR independent. Alternatively, the functional impact of these EVs on endothelial cells is HuR dependent, as demonstrated *in vitro* in endothelial cells treated with EVs, in tumors utilizing the PalmGRET reporter, and in tumors treated intraperitoneally with EVs. It is important to note that EV cargo studies were conducted on a subpopulation of small EVs isolated via SEC, whereas the PalmGRET model tracked all membrane-bound particles. However, administering EVs isolated via SEC into HuR-KO tumors was sufficient to rescue the phenotypes observed in the PalmGRET model, suggesting at least some overlap between the two systems. Although intraperitoneal EV delivery confirmed functional sufficiency, it does not fully mimic endogenous EV transfer and may involve systemic effects. We used this method to ensure consistent exposure, but future studies using physiologic delivery models will be necessary to refine these findings.

Using murine PDAC models, we demonstrated that HuR is necessary for endothelial cell recruitment within the PDAC TME. Re-expression of HuR in HuR-KO cells restored endothelial cell abundance in subcutaneous tumors, indicating that the phenotype is specific to HuR loss rather than off-target effects. Although subcutaneous tumors do not fully reflect the orthotopic TME, particularly in collagen organization that can impact vascular function, this model still serves as a proof of concept that HuR directly regulates endothelial cell levels (58). Our intraperitoneal EV injection rescue experiments provide direct evidence that HuR-dependent EVs increase endothelial cell presence in the TME. Importantly, our mouse model data are concordant with the correlation between *ELAVL1* expression and endothelial cell abundance in patient samples. Future work is warranted to further investigate patient samples to not only gain an understanding from image-based techniques but also include more vascular functional markers and determine whether this correlates with tumor HuR abundance. Of note, vascular function, rather than presence, correlates with patient outcomes (9).

Endothelial cells are dynamic and crucial cells that constitute the barrier of the vasculature throughout the body. As widely reported, tumor vasculature is often dysfunctional, further exacerbating hypoxia within tumors and promoting chaotic angiogenesis (59). In both WT and HuR-KO tumors, we found that only 50% of the vasculature was functional; in WT tumors, there was an increase in DLL4-expressing tip cells. The high level of DLL4-expressing cells indicates that the vasculature is chaotic and uncontrolled (60). This chaotic vasculature may promote metastasis and/or immune evasion in PDAC tumors (9). A key consideration is that some of the observed changes may result from complex cell–cell signaling within the TME. We previously showed that HuR enhances cytokine production, which promotes CAF activation and collagen deposition (16). Given the close connection between stromal remodeling, interstitial pressure, and vascular function, future studies are needed to more fully elucidate the interplay between these processes and how they collectively contribute to the vascular phenotypes observed here (6, 43). To contextualize these findings, we acknowledge that the use of Matrigel in our models may alter the extracellular matrix composition and vascular phenotypes, representing a limitation of the current models.

There is an important interplay between vasculature and immune cells that allows for proper immunosurveillance within tissues (53, 54). During inflammatory responses, endothelial cells express molecules that promote leukocyte transendothelial cell migration, such as ICAM-1, and reduce barrier permeability (61). Our data suggest that in WT tumors, ICAM-1 surface expression is impaired when compared with that in HuR-KO tumors. Importantly, it is specifically the endothelial cells that have imported PDAC EVs in WT tumors that have a decrease in ICAM-1 surface expression compared with endothelial cells within the same tumor that have not imported EVs. The decrease in ICAM-1 surface expression could be associated with HuR-dependent EV cargoes that suppress Wnt or NF-κB signaling, both of which increase ICAM-1 expression in endothelial cells (62, 63). Specifically, protein cargo SOD3, SFRP1, and DKK1 and mRNA cargo *DKK1* were significantly enriched in WT EVs and have been implicated in the suppression of Wnt or NF-κB signaling (64–68). Alternatively, RAC1 protein and *RHOA* mRNA cargo in WT EVs regulate the actin cytoskeleton and may alter junctions between endothelial cells, contributing to the increased sprouting and decreased ICAM-1 observed in WT tumors (69, 70). Although our multiomics analysis identified candidate EV cargo regulated by HuR, we did not functionally validate individual molecules. Thus, the observed endothelial changes likely reflect the combined action of multiple EV components rather than a single effector. Together, these data implicate PDAC EV signaling in regulating endothelial cell ICAM-1 surface expression, which has potential implications for immune trafficking. In fact, our previous study found that HuR-KO tumors have fewer and less activated CD4^+^ T cells (bioRxiv 2025.02.07.632847). Collectively, these results demonstrate that PDAC EV signaling to endothelial cells may, in part, be responsible for increasing CD4^+^ T-cell infiltration into tumors. Future studies are warranted to understand whether vascular remodeling can directly regulate immune infiltration and function in our model.

In conclusion, our findings define HuR as an important regulator of tumor-derived EV cargoes, specifically within the PDAC EV–endothelial cell signaling axis. We showed that WT EVs are tumor promoting and can directly increase endothelial cell migration and tube formation. Moreover, using *in vivo* modeling, we showed that EVs imported by endothelial cells exhibit distinct functional changes within WT tumors. The direct mechanism by which PDAC EVs induce these phenotypes in endothelial cells is most likely due to the many pathways orchestrated by HuR. Overall, our characterization of HuR-dependent EV cargoes and vasculature remodeling within WT tumors contributes to a deeper understanding of the PDAC EV signaling axis and how tumor-intrinsic HuR directly affects the TME. This study provides the groundwork for identifying future vascular targets within the PDAC TME to improve the delivery and efficacy of current therapies targeting PDAC.

## Supplementary Material

Supplementary Figure S1 PANC-1 HuR KO and EV isolation and phospho-proteomics.

Supplementary Figure S2 Validation of EV import imaging in vitro.

Supplementary Figure S3 Repeating in vitro EV treatments with an additional endothelial cell line.

Supplementary Figure S4 Additional mouse model staining.

Supplementary Figure S5 Validation of PalmGRET reporter in KPC cells.

Supplementary Figure S6 EV injected into mice are imported by cells in PDAC tumors.

Supplementary Table S1 Immunoblotting antibody information

Supplementary Table S2 Immunofluorescence antibody information

Supplementary Table S3 Flow cytometry antibody information

Supplementary Table S4 Flow cytometry gating strategy for orthotopic pancreatic tumors

Supplementary Data 1 Supplementary Data 1. WT vs. HuR KO EV cargo

Supplementary Data 2 Supplementary Data 2. Proteomic data from WT vs. HuR KO EV cargo

Supplementary Data 3 Supplementary Data 3. HPaMECs treated with WT vs. HuR KO EVs
