# Supplementary material for: HuR-Regulated Extracellular Vesicles Promote Endothelial Cell Remodeling in Pancreatic Cancer
Source: Cancer Res Commun. 2025 Sep 3;5(9):1501–15. doi: 10.1158/2767-9764.CRC-25-0355 (PMC12405104; doi:10.1158/2767-9764.CRC-25-0355)
Supplement: Supplementary Figure S1 — PANC-1 HuR KO and EV isolation and phospho-proteomics. [file crc-25-0355_supplementary_figure_s1_suppsf1.pdf]

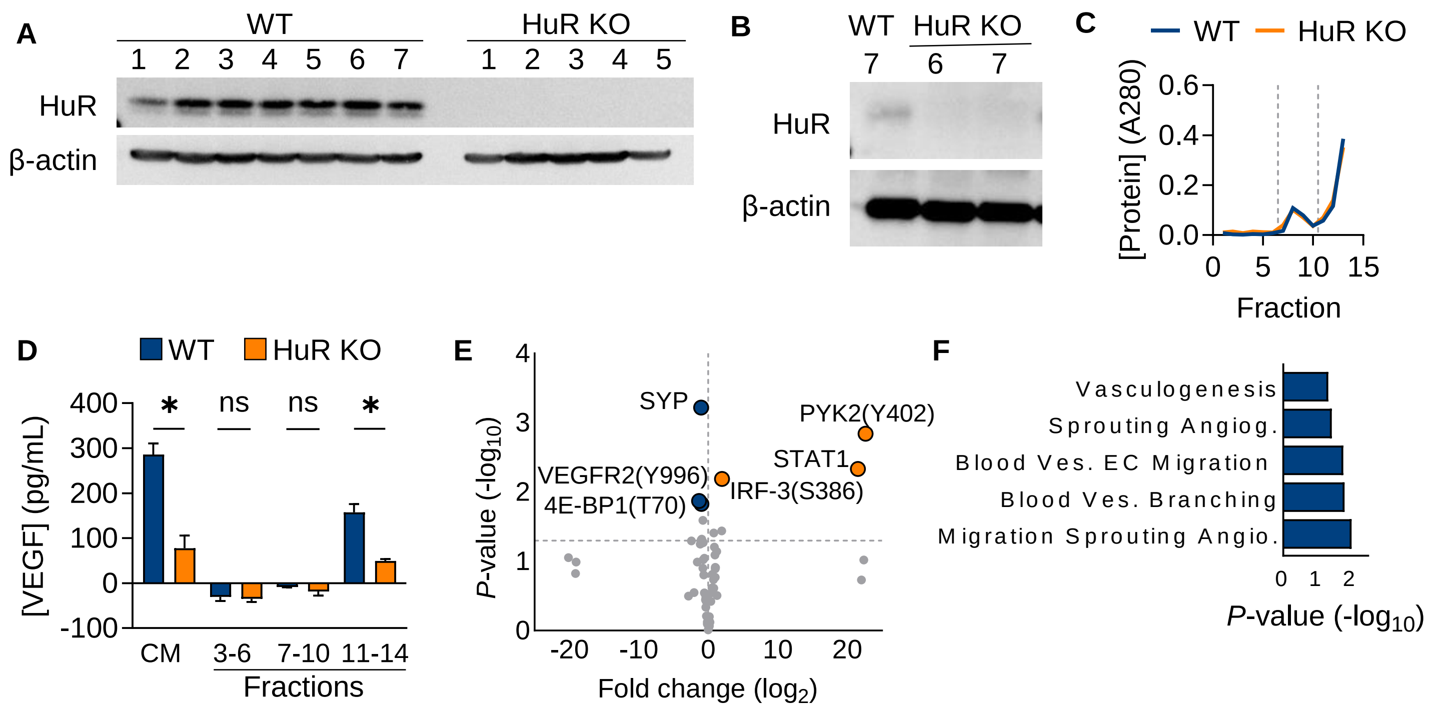

Supplementary Figure S1: PANC-1 HuR KO and EV isolation and phospho-proteomics. A, Immunoblot validation of HuR expression in PANC-1 WT single cell clones 1-7 and HuR KO clones 1-5 probed for HuR and loading control β-actin. **B,** Immunoblot validation of HuR expression in PANC-1 WT single cell clone 7 and HuR KO clones 6-7 probed for HuR and loading control β-actin. **C,** Protein concentration of SEC fractions 1-14 for PANC-1 WT (blue) vs. HuR KO (orange) EV isolations (*n* = 3). **D,** VEGF concentration (pg/mL) via ELISA quantification of PANC-1 WT (blue) vs. HuR KO (orange) conditioned media (CM) and SEC fractions 3-5 (pre-EV), 7-10 (EV containing), and 11-14 (post-EV) (*n* = 3). **E,** Volcano plot of differentially abundant proteins and phospho-proteins (amino acid phosphorylated listed) enriched in WT (left, blue) vs. HuR KO (right, orange) EVs (*n* = 3). **F,** Gene ontology analysis of proteins and phospho-proteins enriched in WT EVs.
