# Supplementary material for: HuR-Regulated Extracellular Vesicles Promote Endothelial Cell Remodeling in Pancreatic Cancer
Source: Cancer Res Commun. 2025 Sep 3;5(9):1501–15. doi: 10.1158/2767-9764.CRC-25-0355 (PMC12405104; doi:10.1158/2767-9764.CRC-25-0355)
Supplement: Supplementary Figure S2 — Validation of EV import imaging in vitro. [file crc-25-0355_supplementary_figure_s2_suppsf2.pdf]

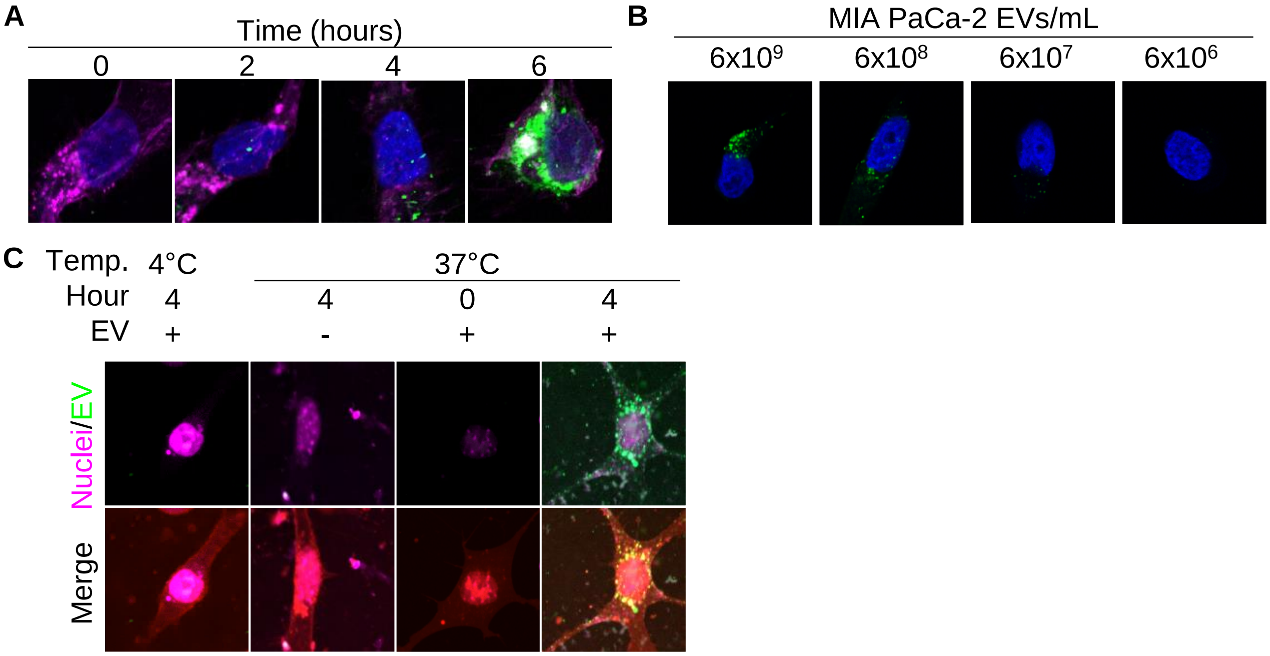

Supplementary Figure S2: Validation of EV import imaging *in vitro*. A, MIA PaCa-2 cells treated with PKH67 labeled MIA PaCa-2 EVs (green) and collected at 0-6 hours and stained for nuclei (DAPI, blue) and the cell surface (wheat germ agglutinin, magenta). **B,** MIA PaCa-2 cells treated with a titration of 6×10^6 – 6×10^9 PKH67-labeled MIA PaCa-2 EVs (green) and collected at 4 hours and stained for nuclei (DAPI, blue). **C,** Cancer associated fibroblasts treated with PKH67 labeled MIA PaCa-2 EVs (green) for 0 and 4 hours at 37°C and 4°C or PKH67 alone (EV-) for 4 hours.
