# Supplementary material for: HuR-Regulated Extracellular Vesicles Promote Endothelial Cell Remodeling in Pancreatic Cancer
Source: Cancer Res Commun. 2025 Sep 3;5(9):1501–15. doi: 10.1158/2767-9764.CRC-25-0355 (PMC12405104; doi:10.1158/2767-9764.CRC-25-0355)
Supplement: Supplementary Figure S3 — Repeating in vitro EV treatments with an additional endothelial cell line. [file crc-25-0355_supplementary_figure_s3_suppsf3.pdf]

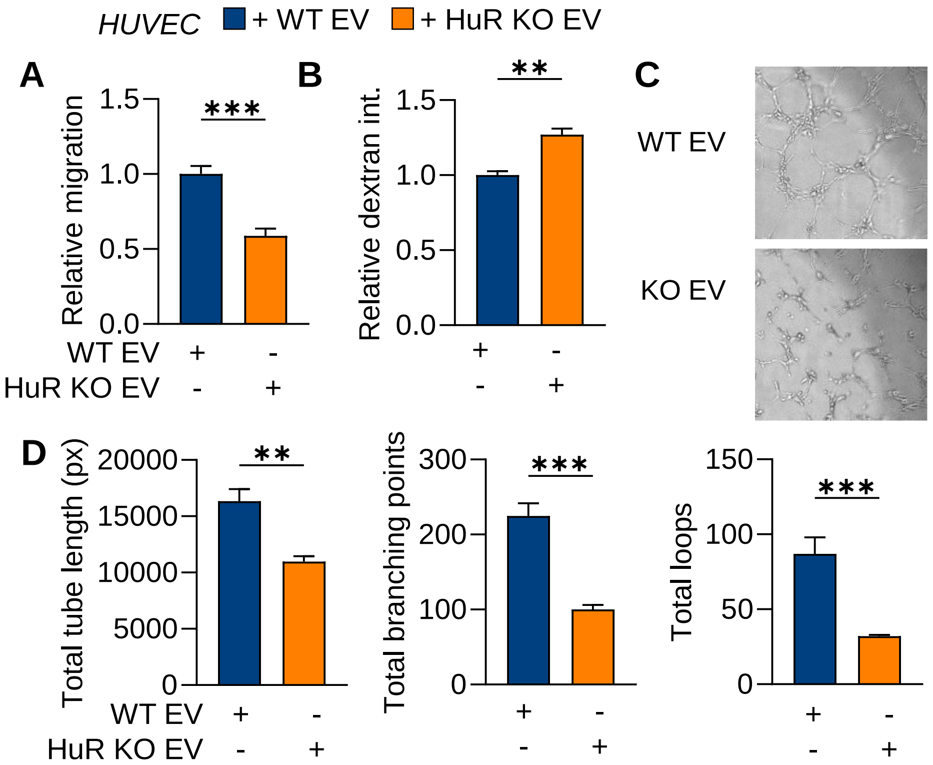

Supplementary Figure S3: Repeating *in vitro* EV treatments with an additional endothelial cell line. A, Transwell migration of HUVECs when treated with PANC-1 WT vs. HuR KO EVs over 24 hours and quantified with crystal violet staining (*n* = 3). Functional analysis of HUVECs treated with media alone, PANC-1 WT or HuR KO EVs for 24 hours and monitored for **B,** monolayer permeability quantified by dextran movement across the endothelial cell monolayer and **C,** tube formation quantified for **D,** total tube length (px), total branching points and total loops (*n* = 3). *P* values were calculated using an unpaired two-tailed Student's *t*-test. *, $P < 0.05$; **, $P < 0.01$; ***, $P < 0.001$; ns, not significant.
