# Supplementary material for: HuR-Regulated Extracellular Vesicles Promote Endothelial Cell Remodeling in Pancreatic Cancer
Source: Cancer Res Commun. 2025 Sep 3;5(9):1501–15. doi: 10.1158/2767-9764.CRC-25-0355 (PMC12405104; doi:10.1158/2767-9764.CRC-25-0355)
Supplement: Supplementary Figure S4 — Additional mouse model staining. [file crc-25-0355_supplementary_figure_s4_suppsf4.pdf]

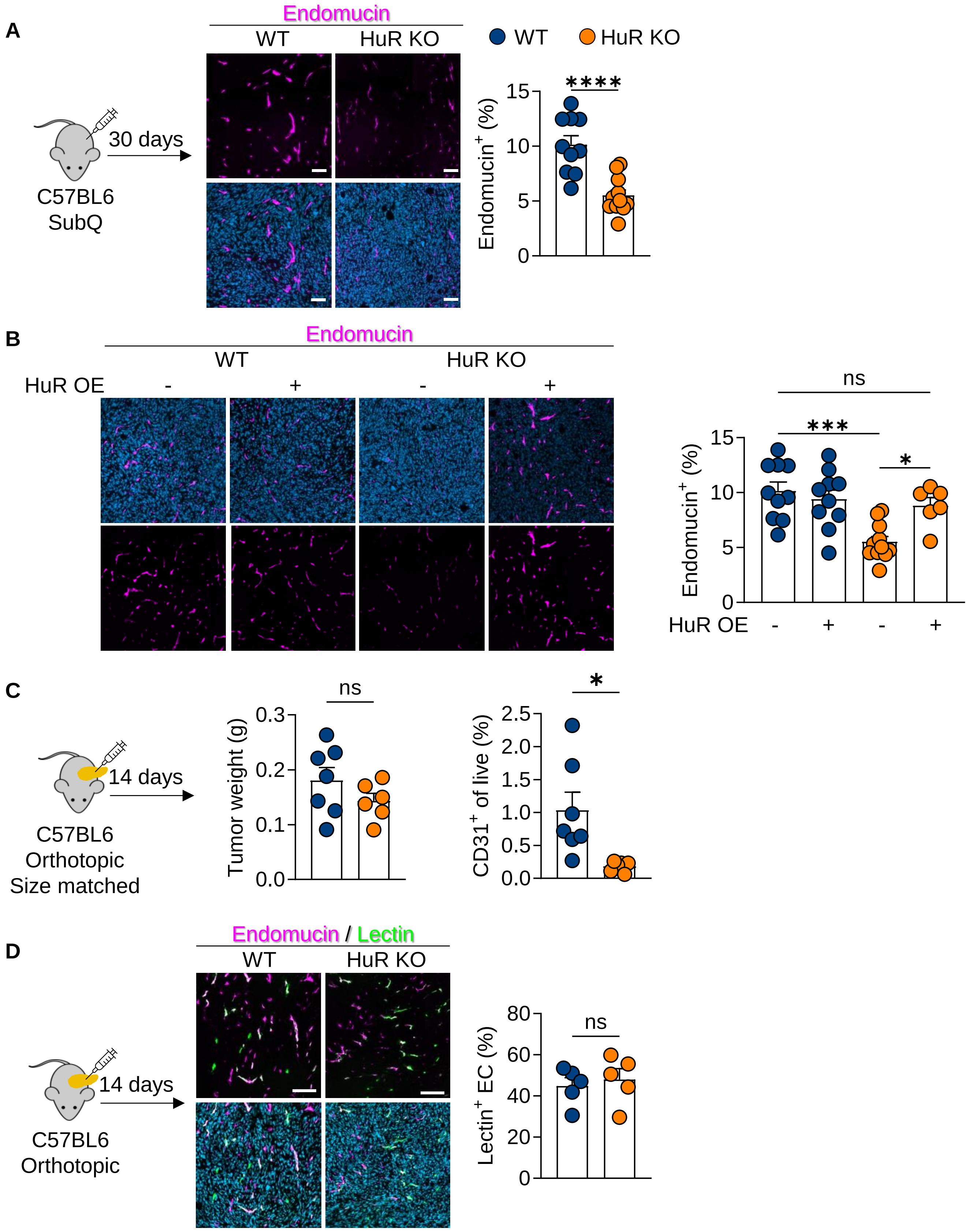

Supplementary Figure S4: Additional mouse model staining. A, KPC WT vs. HuR KO cells subcutaneously implanted into the flank of C57BL6 mice and euthanized after 30 days and stained for endomucin (magenta) and nuclei (DAPI, blue) (n = 10). **B,** KPC WT and HuR KO cells with retroviral re-expression (OE) of HuR were implanted into the flank of C57BL6 mice and euthanized after 30 days and stained for endomucin (magenta) and

nuclei (DAPI, blue) (WT, *n* = 10; WT + OE, *n* = 10; KO, *n* = 11; KO + OE, *n* = 6). **C,** Tumor volume after 14 days post orthotopic pancreatic injection of $4x10^5$ KPC WT or $1.6x10^6$ HuR KO cells were implanted orthotopically into C57BL6 mice. Percentage of endothelial cells ($CD45^-CD31^+$) cells were quantified via flow cytometry on dissociated tumors (*n* = 7). **D,** Orthotopic WT vs. HuR KO tumors perfused with lectin (green) and stained for nuclei (DAPI, blue) and endothelial cells (endomucin, magenta) (*n* = 5). Scale bars = 100 µm. *P* values were calculated using an unpaired two-tailed Student's *t*-test or an ordinary one-way ANOVA (panel B only) *, *P* < 0.05; **, *P* < 0.01; ***, *P* < 0.001; ns, not significant.
