# Supplementary material for: HuR-Regulated Extracellular Vesicles Promote Endothelial Cell Remodeling in Pancreatic Cancer
Source: Cancer Res Commun. 2025 Sep 3;5(9):1501–15. doi: 10.1158/2767-9764.CRC-25-0355 (PMC12405104; doi:10.1158/2767-9764.CRC-25-0355)
Supplement: Supplementary Figure S5 — Validation of PalmGRET reporter in KPC cells. [file crc-25-0355_supplementary_figure_s5_suppfs5.pdf]

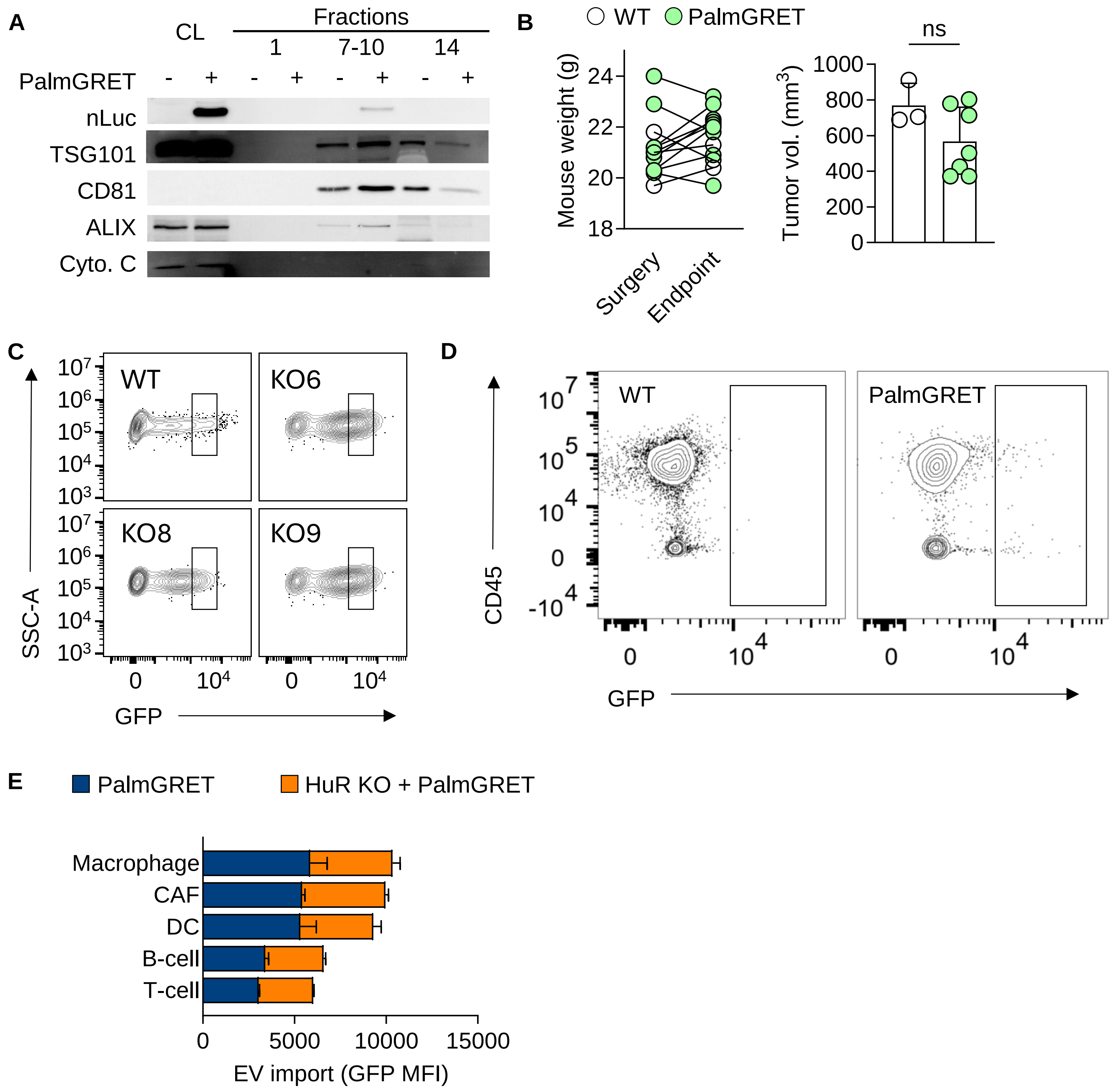

Supplementary Figure S5: Validation of PalmGRET reporter in KPC cells. A, Immunoblot of cell lysates (CL) and SEC fractions 1, 7-10 (EV) and 14 from KPC WT and PalmGRET cells and probed for the PalmGRET reporter expression (nLuc), classical EV markers (TSG101, CD81, ALIX) and negative control cytochrome C. **B,** Mouse bodyweight and tumor volume from KPC WT (n = 3) and PalmGRET (n = 7) orthotopic tumors after 21 days post implantation. **C,** Flow contour plots of transduced KPC cells with PalmGRET with gates for fluorescence-activated cell sorting based on GFP intensity. **D,** Flow contour plots of flow cytometry staining controls relating to Figure 5E. WT tumors were stained and utilized as the negative control for GFP compared to a mix of all PalmGRET tumors (WT and HuR KO). **E,** EV import across all stromal cells in flow cytometry panel in PalmGRET vs. PalmGRET HuR KO tumors as reported by GFP geometric mean (MFI). P values were calculated using an unpaired two-tailed Student's t-test. *, $P < 0.05$; **, $P < 0.01$; ***, $P < 0.001$; ns, not significant.
