# Supplementary material for: HuR-Regulated Extracellular Vesicles Promote Endothelial Cell Remodeling in Pancreatic Cancer
Source: Cancer Res Commun. 2025 Sep 3;5(9):1501–15. doi: 10.1158/2767-9764.CRC-25-0355 (PMC12405104; doi:10.1158/2767-9764.CRC-25-0355)
Supplement: Supplementary Figure S6 — EV injected into mice are imported by cells in PDAC tumors. [file crc-25-0355_supplementary_figure_s6_suppsf6.pdf]

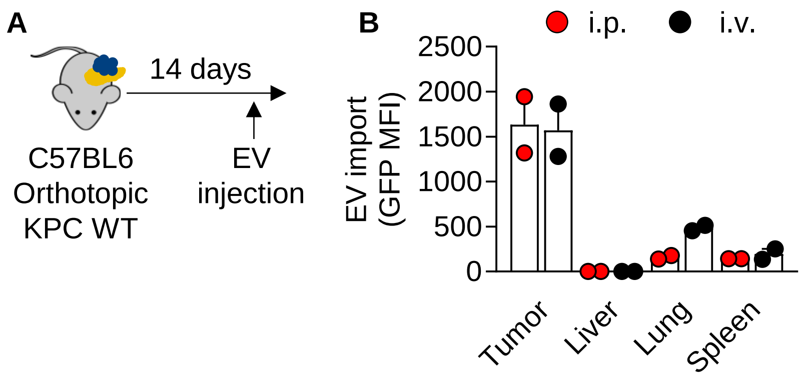

Supplementary Figure S6: EV injected into mice are imported by cells in PDAC tumors. A, Immunocompetent mice were orthotopically injected with KPC WT cells. After 14 days PKH67 labeled EVs were injected i.p. (red) or i.v. (black) into mice. **B,** Tissues were dissociated and EV import was assessed via flow cytometry (*n* = 2).
