# Supplementary material for: HuR-Regulated Extracellular Vesicles Promote Endothelial Cell Remodeling in Pancreatic Cancer
Source: Cancer Res Commun. 2025 Sep 3;5(9):1501–15. doi: 10.1158/2767-9764.CRC-25-0355 (PMC12405104; doi:10.1158/2767-9764.CRC-25-0355)
Supplement: Supplementary Table S1 — Immunoblotting antibody information [file crc-25-0355_supplementary_table_s1_suppst1.pdf]

Supplementary Table S1: Immunoblotting antibody information			
**Marker**	**Source**	**Catalog #**	**RRID**
HuR	Santa Cruz	sc-5261	RRID:AB_627770
Vinculin	Santa Cruz	sc-73614	RRID:AB_113129
TSG101	Abcam	ab125011	RRID:AB_10974262
CD81	Santa Cruz	sc-166029	RRID:AB_2275892
ALIX	Cell Signaling	2171	RRID:AB_2299455
Cytochrome C	Cell Signaling	11940	RRID:AB_2637071
β-actin	Cell Signaling	4967S	RRID:AB_330288
nLuc	Promega	N7000	RRID:AB_3095534
