# Supplementary material for: HuR-Regulated Extracellular Vesicles Promote Endothelial Cell Remodeling in Pancreatic Cancer
Source: Cancer Res Commun. 2025 Sep 3;5(9):1501–15. doi: 10.1158/2767-9764.CRC-25-0355 (PMC12405104; doi:10.1158/2767-9764.CRC-25-0355)
Supplement: Supplementary Table S2 — Immunofluorescence antibody information [file crc-25-0355_supplementary_table_s2_suppst2.pdf]

| Supplementary Table S2: Immunofluorescence antibody information | | | |
|---|---|---|---|
| **Marker** | **Source** | **Catalog #** | **RRID** |
| Ki67 | Cell Signaling | 12202 | RRID:AB_2620142 |
| Endomucin | eBiosciences | 14-5851-82 | RRID:AB_891527 |
| DLL4 | Novus Biologicals | AF1389-SP | RRID:AB_354770 |
| ICAM-1 | Thermo Scientific | 14-0541-82 | RRID:AB_467301 |
| CD31 | Cell Signaling | 77699 | RRID:AB_2722705 |
