# Supplementary material for: HuR-Regulated Extracellular Vesicles Promote Endothelial Cell Remodeling in Pancreatic Cancer
Source: Cancer Res Commun. 2025 Sep 3;5(9):1501–15. doi: 10.1158/2767-9764.CRC-25-0355 (PMC12405104; doi:10.1158/2767-9764.CRC-25-0355)
Supplement: Supplementary Table S3 — Flow cytometry antibody information [file crc-25-0355_supplementary_table_s3_suppst3.pdf]

Supplementary Table S3: Flow cytometry antibody information					
**Marker**	**Fluorophore**	**Dilution**	**Source**	**Catalog #**	**RRID**
MHC-II	AF700	1:100	BioLegend	107622	RRID:AB_493727
ICAM-1	APC-Fire750	1:100	BioLegend	116126	RRID:AB_2716074
CD19	BUV805	1:200	BD	568287	RRID:AB_2916875
CD90.2	BUV395	1:100	BD	565257	RRID:AB_2739136
Live/Dead	LD Blue	1:500	Invitrogen	L23105	NA
F4/80	BV421	1:100	BioLegend	123137	RRID:AB_2563102
CD11c	BV650	1:100	BioLegend	117339	RRID:AB_2562414
CD31	BV785	1:100	BioLegend	102435	RRID:AB_2810334
PDPN	PE	1:100	BioLegend	127408	RRID:AB_2161928
CD45.2	PE Cy7	1:100	BioLegend	109830	RRID:AB_1186098
