# Supplementary material for: HuR-Regulated Extracellular Vesicles Promote Endothelial Cell Remodeling in Pancreatic Cancer
Source: Cancer Res Commun. 2025 Sep 3;5(9):1501–15. doi: 10.1158/2767-9764.CRC-25-0355 (PMC12405104; doi:10.1158/2767-9764.CRC-25-0355)
Supplement: Supplementary Table S4 — Flow cytometry gating strategy for orthotopic pancreatic tumors [file crc-25-0355_supplementary_table_s4_suppst4.pdf]

Supplementary Table S4: Flow cytometry gating strategy for orthotopic pancreatic tumors	
**Cell Type**	**Gating (of live single cells)**
EC	$CD45^{-}CD31^{+}$
CAF	$CD45^{-}CD31^{-}PDPN^{+}$
T cell	$CD45^{+}MHCII^{-}CD90^{+}$
B cell	$CD45^{+}MHCII^{+}CD19^{+}$
DC	$CD45^{+}MHCII^{+}CD11c^{+}$
Macrophage	$CD45^{+}MHCII^{+}F4/80^{+}$
